# Verbal memory depends on structural hippocampal subfield volume

**DOI:** 10.3389/fneur.2023.1209941

**Published:** 2023-10-13

**Authors:** Panagiota-Eleni Tsalouchidou, Christina-Julia Müller, Marcus Belke, Felix Zahnert, Katja Menzler, Eugen Trinka, Susanne Knake, Aljoscha Thomschewski

**Affiliations:** ^1^Epilepsy Center Hessen, Department of Neurology, Philipps University Marburg, Marburg, Germany; ^2^Center for Personalized Translational Epilepsy Research (CePTER), Frankfurt, Germany; ^3^Department of Neurology and Centre for Cognitive Neuroscience, Christian Doppler University Hospital, Paracelsus Medical University, Member of the European Reference Network EpiCARE, Salzburg, Austria; ^4^Neuroscience Institute, Christian Doppler University Hospital, Paracelsus Medical University, Salzburg, Austria

**Keywords:** hippocampus, temporal lobe epilepsy, mild cognitive impairment, hippocampal subfields, subiculum, CA2/CA3

## Abstract

**Objective:**

To investigate correlates in hippocampal subfield volume and verbal and visual memory function in patients with temporal lobe epilepsy (TLE), mild amnestic cognitive impairment (MCI) and heathy participants (HP).

**Methods:**

50 right-handed participants were included in this study; 11 patients with temporal lobe epilepsy (TLE), 18 patients with mild amnestic cognitive impairment (MCI) and 21 healthy participants (HP). Verbal memory performance was evaluated via the verbal memory test (VLMT) and visual memory performance via the diagnosticum for cerebral damage (DCM). Hippocampal subfield volumes of T1-weighted Magnetic Resonance Imaging (MRI) scans were computed with FreeSurfer version 7.1. Stepwise correlation analyses were performed between the left hippocampal subfield volumes and learning, free recall, consolidation and recognition performance scores of the VLMT as well as between right hippocampal subfield volumes and visual memory performance.

**Results:**

The volume of the left subicular complex was highly correlated to learning performance (*β* = 0.284; *p* = 0.042) and free recall performance in the VLMT (*β* = 0.434; *p* = 0.001). The volume of the left CA3 subfield showed a significant correlation to the consolidation performance in the VLMT (*β* = 0.378; *p* = 0.006) and recognition performance in the VLMT (*β* = 0.290; *p* = 0.037). There was no significant correlation identified between the right hippocampal subfields and the visual memory performance.

**Conclusion:**

The results of this study show verbal memory correlates with hippocampal subfields and support the role of left subiculum and left CA2/CA3 in verbal memory performance.

## 1. Introduction

The hippocampus is a structurally and functionally complex brain structure which is mainly involved in episodic, semantic and spatial memory processes (1). The dominant, most often left hippocampus is mostly involved in the verbal memory performance while the non-dominant, right hippocampus is involved in spatial and visual memory performance (2–4). Although the underlying pathomechanisms differ, the hippocampal formation and its subfields are affected in many neurological and psychiatric conditions such as Alzheimer disease (AD), mild amnestic cognitive impairment (MCI), schizophrenia, temporal lobe epilepsy (TLE) as well as in normal aging, causing visual and verbal memory decline (5–11).

Histopathological and neuroimaging studies have demonstrated correlations between memory performance with neural density and volumes of hippocampal substructures (5–8). The evolution of high-field brain magnetic resonance imaging (MRI) and advanced automatic segmentation techniques enable the accurate measurement of hippocampal subfield volumes (12) and subsequently *in vivo* correlations of memory performance with hippocampal atrophy patterns (13). In the present study, it was our aim to identify neuropsychological specific rather than disease-specific correlations between verbal and non-verbal memory performance and hippocampal subfield volumes. Therefore, we conducted the analyses in different populations known to develop deficits in verbal and non-verbal memory function such as in patients with temporal lobe epilepsy (TLE) or mild cognitive impairment (MCI) as well as in elderly healthy participants (HP).

## 2. Materials and methods

### 2.1. Study population and design

Eleven patients with temporal lobe epilepsy (TLE), 18 patients with mild cognitive impairment (MCI) and 21 healthy participants (HP) were included in the study. All participants were recruited in the Department of Neurology, Christian Doppler University Hospital Salzburg, Austria and received a multimodal neuropsychological evaluation and a three-dimensional (3D), T1-weighted MRI scan. The TLE diagnosis was based on neurological assessment by experienced epileptologists, including video-EEG examination for up to five days. There was no evidence of hippocampal sclerosis in the included TLE population. The MCI diagnosis was based on Petersen’s criteria (14). All MCI patients reported subjective amnestic complaints corresponding to the level three of the global deterioration scale for aging and dementia (15). All TLE and MCI patients did not have any neuropsychiatric comorbidities and all recruited HP did not have any history of neurological or psychiatric diseases. From the initial sample, four participants were excluded from the analysis due to motion artifacts on the MRI scan. Two left-handed subjects were excluded from the analysis to avoid atypical hemispheric representation (16). One additional participant did not agree to submit neuropsychological data and two participants did not complete the DCS, leaving a sample size of a total of 50 participants. The study was approved by the local ethics committee (Ethics Commission Salzburg/Ethikkommission Land Salzburg; approval number 415-E/1429) and all participants gave written informed consent.

### 2.2. Neuropsychological evaluation

All participants underwent a multimodal neuropsychological evaluation including verbal and non-verbal memory performance assessment using the verbal memory test (VLMT) (17) and the diagnosticum for cerebral damage (DCM) (18), respectively. The T-values of the four scales of VLMT in learning performance, free recall, consolidation performance and recognition were entered in the analysis. Here, a list of 15 nonrelated words is initially verbally presented by the examiner five times. The participant is asked each time to repeat as many words as possible. The correctly recalled words represent the learning performance of the VLMT. Afterwards, an interference list of 15 words is verbally presented by the examiner and the participant is asked to recall the words of the first list. Without any other presentation, the participant is asked to recall the initial list with a 30-min delay which refers to free recall performance. The consolidation performance represents the difference of the recalled words after the 30-min delay in relation to the number of words remembered after the fifth repetition of the initial list presentation. Finally, the recognition performance refers to the ability of the participant to recognize the words of the first list from a larger list of verbally presented words. For the non-verbal performance, the percentile summary score of the DCM was entered in the analysis.

### 2.3. MRI acquisition

All three-dimensional (3D), T1-weighted MRI scans of the participants were performed with a 3T Siemens (Erlangen, Germany) Magnetom TrioTim syngo MR B17 scanner, a 12-channel head coil and the following parameters: sagittal orientation, 192 slices per slab, 256 mm FoV read at 93.8%phase, voxel dimension 1 × 1 × 1 mm, repetition time (TR) 2,300 ms, echo time (TE) 2.91 ms, inversion time (TI) 900 ms, flip angle (FA) 9°.

### 2.4. Estimation of the hippocampal subfields and image quality control

The hippocampal subfield volumes were computed with the open-source software FreeSurfer version 7.1 (v.7.1)^1^ (19, 20). Briefly, each 3D T1-weighted scan was pre-processed using the FreeSurfer recon-all script, which automatically generated a surface reconstruction and segmentation.^2^ The hippocampal subfield volumes were computed with the subfield segmentation pipeline (12), which can be found at https://surfer.nmr.mgh.harvard.edu/fswiki/HippocampalSubfields. The detailed output volumes are presented at https://surfer.nmr.mgh.harvard.edu/fswiki/HippocampalSubfieldsAndNucleiOfAmygdala. The pipeline shows a good test–retest reliability and reliability across vendor platforms and field-strengths and has been applied in several neurocognitive studies, which examine hippocampal pathologies including patients with TLE and MCI (21). All raw MR data were controlled for quality and motion artifacts before segmentation. Inspection of all the cortical and subcortical results after the general segmentation with the recon-all script and inspection of the results after the subfield segmentation were performed in each step, respectively. No manual correction was required, after visual inspection of each output for significant segmentation errors.

### 2.5. Correction for total brain volume

All subfield volumes were corrected for total brain volume before the analysis. For the correction, we used a covariance approach (13, 22, 23) using a normal collective of 256 healthy subjects, who received 3D T1-weighted MPRAGE scans (FOV 256 × 256 × 176 voxel, voxel dimension 1 × 1 × 1 mm, TR 1900 ms, TE 2.52 ms, TI 900 ms. FA 9°, BW 170 Hz/pixel) on a 3T Trio scanner (Siemens, Erlangen, Germany) at the Center for Brain Imaging in Marburg, Germany. Briefly, we calculated the slope β of the linear regression between each structure and the total brain volume for all control subjects of the normal collective. The correction of each volume was computed using the following equation: Volcorr = Volorig − β (TBVorig − TBVmean) in which Volcorr is the corrected volume of the structure, Volorig is the original volume of the structure, β is the slope of the linear regression, TBVorig is the original total brain volume of the subject and TBVmean is the mean total brain volume for all control subjects. For the total brain volume (TBVorig), the measure “BrainSegVolNotVent” in the FreeSurfer segmentation output was used. The segmentation results were corrected for total brain volume to minimize the effect of age and gender (24, 25).

#### 2.5.1. Statistical analysis

In the next step, the relationship between corrected subfield volumes and neuropsychological variables was examined. The analyses were performed with the IBM SPSS Statistics program version 26.0. To determine whether the volumes of certain hippocampal subfields were related to neuropsychological outcomes, regression analyses were computed separately for the left and right hippocampal subfield volumes. To avoid problems of multicollinearity, stepwise regression methods were used in entering the predictors. For verbal memory, T-values of learning performance, free recall, consolidation performance and recognition of the VLMT were used as outcome variables and all subfields of the left hippocampi were entered into the regression analyses as predictors. For figural memory, the percentile ranks of the DCS served as outcome measure and all subfield volumes of the right hippocampi were entered into the regression analysis as predictors. The following subfields were entered into regression analyses: subiculum, presubiculum, and parasubiculum added to subicular complex (SUB) (8, 26), cornu ammonis 1 (CA1), cornu ammonis 2/3 (CA2/CA3), cornu ammonis 4 (CA4), granule cells in the molecular layer of the dentate gyrus (GC-ML-DG), molecular layer, hippocampal amygdala transition area (HATA), fimbria, hippocampal tail and hippocampal fissure. One-way analysis of variance (ANOVA) was used to compare the age among groups.

### 2.6. Data availability statement

Anonymized data can be made available to any qualified investigator upon request.

## 3. Results

### 3.1. Study population, demographics, and clinical characteristics

A total of 50 participants (58% women, *n* = 29) was included in this study. The age of the participants was significantly different among groups [*F*(2, 47): 8.01; *p* < 0.001], primarily due to the younger age of TLE patients. The median disease duration of epileptic seizures was 16 years (range 3–51 years) in the TLE group, and the median duration of amnestic symptoms was 12 months (range 6–24 months) in the MCI group. The patients’ characteristics are presented in Table 1.

**Table 1:** Demographics of the study population.

Sample	All participants	TLE	MCI	HP
N	50	11	18	21
Median age (years)	60	49	66.5	61
Age range (years)	23-76	26–59	50-76	23-74
N women	29	8	9	12

N, number; TLE, temporal lobe epilepsy; MCI, mild cognitive impairment; HP, healthy participants.

### 3.2. Relation of hippocampal subfield volumes to neuropsychological data

#### 3.2.1. Verbal memory

A stepwise linear regression was computed using T-values of the learning performance as outcome variables and subfields of the left hippocampi as predictor variables. The regression model was statistically significant [*R^2^* = 0.08 (adjusted *R^2^* = 0.062), *F*(1, 50) = 4.37; *p* = 0.042]. The volume of the left subicular complex was a significant predictor of learning performance in the VLMT (*β* = 0.284; *p* = 0.042) while no other subfield volumes were significant predictors (*p*s > 0.369).

The regression model using T-values of the free recall performance as outcome variable and subfields of the left hippocampi as predictor variables was statistically significant [*R^2^* = 0.189 (adjusted *R^2^* = 0.172), *F*(1, 50) = 11.61; *p* = 0.001]. The volume of the left subicular complex was a significant predictor of free recall performance in the VLMT (*β* = 0.434; *p* = 0.001) while no other subfield volumes were significant predictors (*p*s > 0.112).

A stepwise linear regression using T-values of the consolidation performance as outcome variable and subfields of the left hippocampi as predictor variables was statistically significant [*R^2^* = 0.143 (adjusted *R^2^* = 0.126), *F*(1, 50) = 8.34; *p* = 0.006]. The volume of the left CA2/CA3 subfield was a significant predictor of consolidation performance in the VLMT (*β* = 0.378; *p* = 0.006) while no other subfield volumes were significant predictors (*p*s > 0.177).

**Figure 1:**
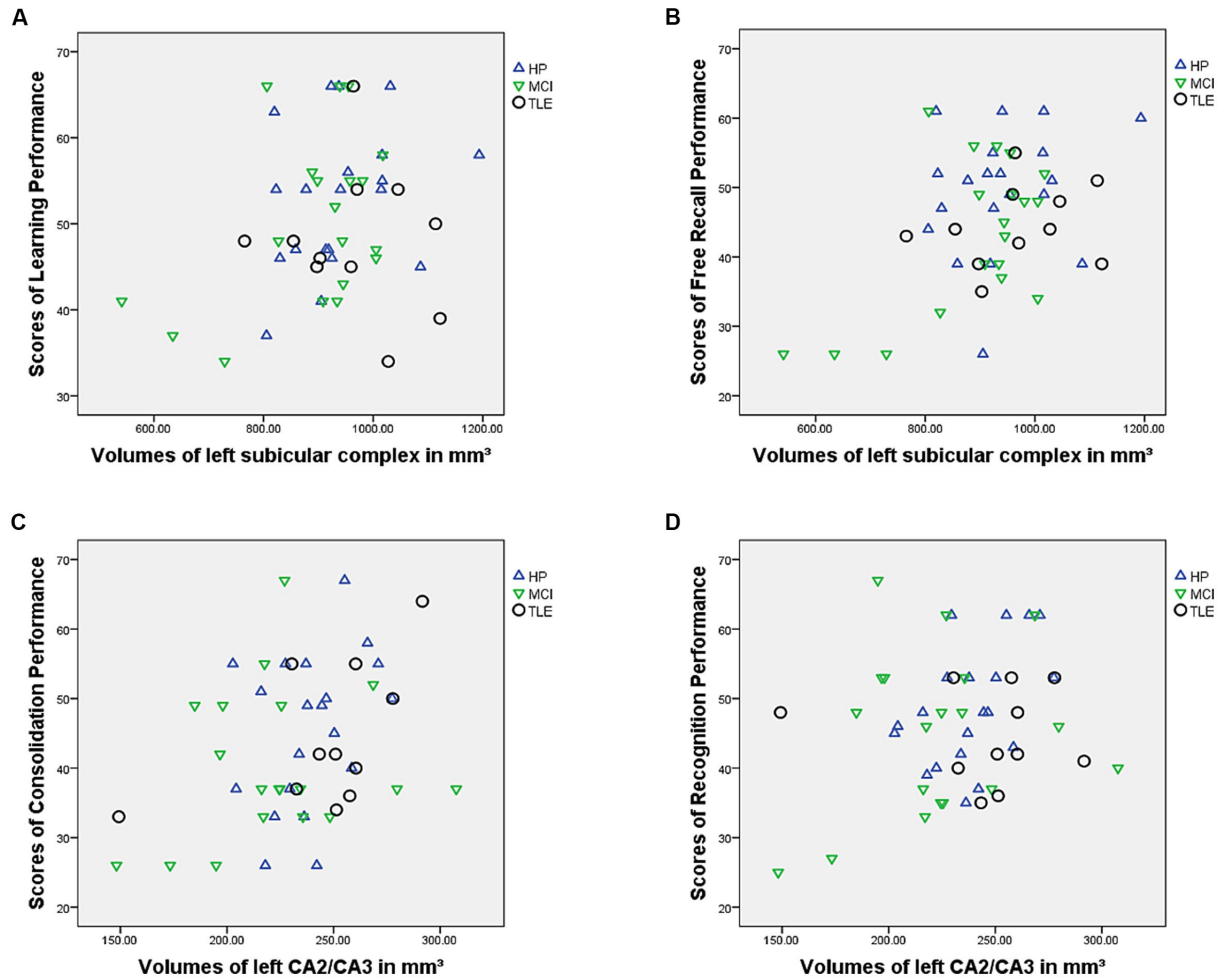
Figure showing relationship between **(A)** learning performance and subicular complex volumes, **(B)** free recall performance and subicular complex volumes, **(C)** consolidation performance and CA2/CA3 volumes and **(D)** recognition performance and CA2/CA3 volumes.

**Figure 2:**
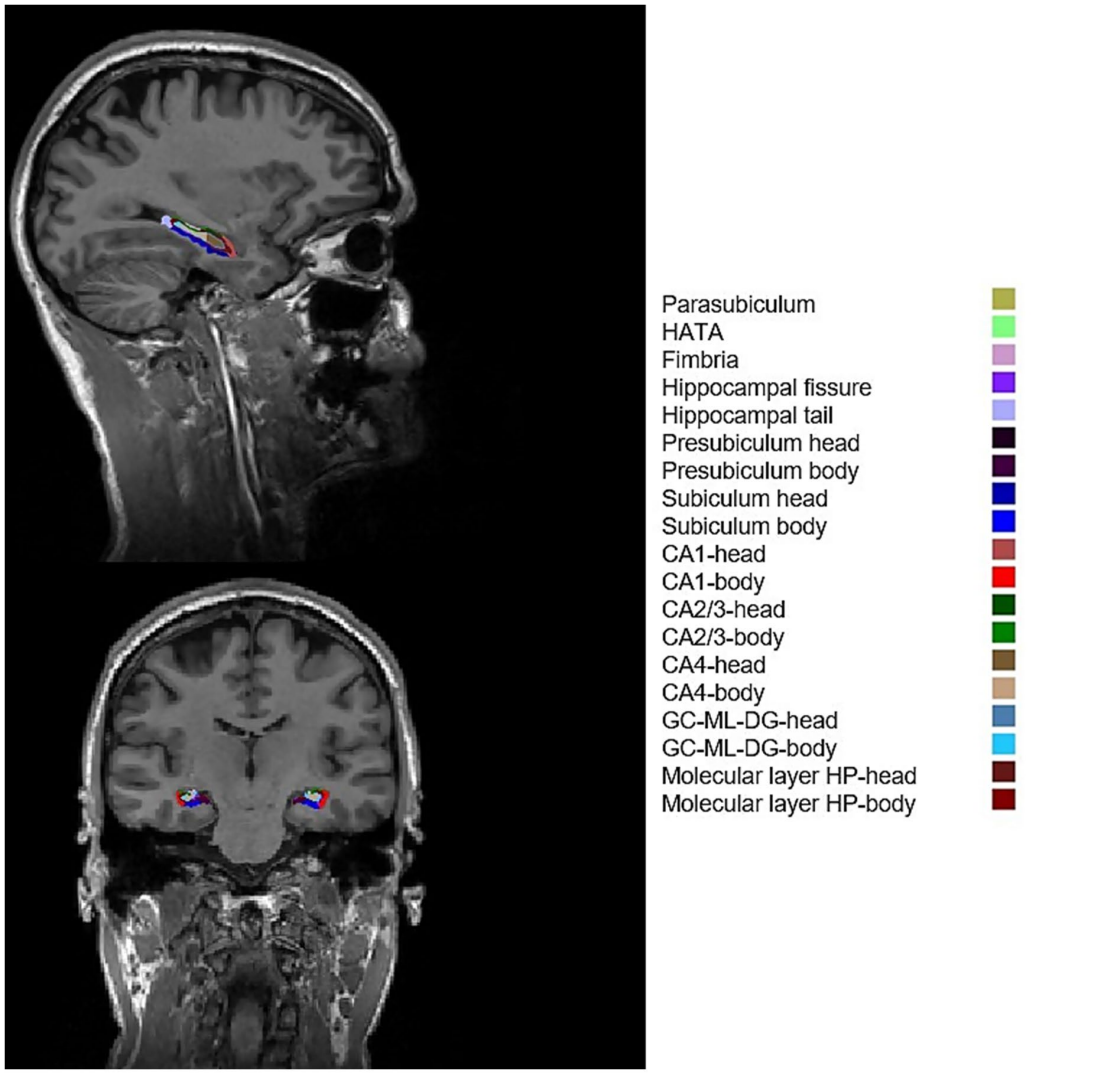
Example of FreeSurfer subfield segmentation results of a healthy participant. CA1, cornu ammonis 1; CA2/3, cornu ammonis 2/3; CA4, cornu ammonis 4; GC-ML-DG, granule cells in the molecular layer of the dentate gyrus; HATA, hippocampal amygdala transition area; HP, hippocampal.

Finally, the regression model using T-values of the recognition performance as outcome variable and subfields of the left hippocampi as predictor variables was statistically significant [*R^2^* = 0.084 (adjusted *R^2^* = 0.066), *F*(1, 50) = 4.59; *p* = 0.037]. The volume of the left CA2/CA3 subfield was a significant predictor of recognition performance in the VLMT (*β* = 0.290; *p* = 0.037) while no other subfield volumes were significant predictors (*p*s > 0.284) (Figures 1, 2).

#### 3.2.2. Figural memory

A stepwise linear regression was computed using percentage rank of the non-verbal memory test DCS as outcome variable and subfields of the right hippocampi as predictors. The regression model was not significant.

## 4. Discussion

The development of more accurate measurement methods of hippocampal subfield volumes enables the *in vivo* identification of neuroanatomical correlates of specific memory functions. In this study, participants with reduced left subicular complex volumes showed significantly reduced learning and 30-min free recall performance. Left subiculum volumes have already been associated with verbal memory functions (7, 10, 27–29). Previous studies have shown that selective subiculum atrophy patterns are correlated with the immediate and delayed recall performance of MCI and Alzheimer patients (7, 27) and can be considered as a marker of conversion to early stages of Alzheimer disease (7, 30, 31). Similar findings in elderly healthy populations and epilepsy patients have demonstrated an association between reduced subiculum volumes and verbal free recall performance decline (10, 28, 29).

The subiculum is considered the main output formation of the hippocampus with projections to several cortical and subcortical regions such as prefrontal and entorhinal cortex, amygdala, nucleus accumbens and hypothalamus, receiving input mainly from CA1 and the entorhinal cortex (26). Due to these extensive connections, the presubiculum and subiculum are considered to be mostly involved in memory retrieval rather than in memory encoding (32, 33). During short memory retrieval, the subiculum has been shown to be activated before the hippocampus, which indicates its role in the retrieval of recently acquired information (34, 35).

Reduced CA2/CA3 volumes were significantly correlated with lower consolidation and recognition performance, while there were no associations identified between DG and CA1 volumes and the four scales of VLMT. The results of previous studies concerning the above mentioned subfields vary; Mueller et al. (36) have previously shown associations of CA3/dentate gyrus (DG) with encoding/early retrieval and CA1 with consolidation performance and late retrieval. Coras et al. (6) have demonstrated significant correlations of declarative memory performance and neural density of CA3/CA4 or DG and less to CA1. Other studies on patients with selective CA3 atrophy caused by leucine-rich glycine-inactivate-1 antibody-complex limbic encephalitis (LGI1-antibody-complex LE) have shown significant associations with CA3 atrophy and impaired recent and remote autobiographical episodic memory. Although autobiographical episodic memory has not been tested by the VLMT in this study population, these results indicate the role of CA3 in episodic memory consolidation (37, 38). Finally, controversial findings have also been shown regarding the role of CA1 in verbal memory performance; some studies have reported correlations between CA1 neural density (39) and volumes (28, 36) with immediate and delayed verbal memory recall as well as long-term memory consolidation (8), while other studies have shown opposite findings (6, 7).

Concerning the figural memory, there were no significant correlates with the right hippocampal subfields and the non-verbal memory test DCS. Previous studies have demonstrated correlations of figural memory with different hippocampal subfield volumes and visual memory recall such as presubiculum (40), CA4 (41), subiculum and CA1 volumes (28). The discrepancy of the results in the above-mentioned studies indicates the need of larger scale samples to further investigate figural memory correlates.

This study has some limitations. First, although high resolution MRI and advanced segmentation methods have contributed to greater accuracy in measuring the hippocampal subfield volumes, these might differ from the real subject’s subfield volumes. Moreover, the results should be interpreted with caution due to the small sample size, different age range and disease duration of the included patients. Finally, we aimed to study a mixed population in order to identify non-disease specific but rather neuropsychological specific correlates; however, the different disease pathomechanisms might influence the hippocampal subfield volumes differently, possibly affecting the correlations identified.

## 5. Conclusion

The findings of this study support previous evidence that hippocampal subfields play an important role in specific memory functions. Identifying neuroanatomical correlates of verbal memory can be useful for applying more targeted therapies. In epilepsy patients, for instance, techniques like stereo-encephalography and highly selective laser surgery techniques might contribute to preserving unaffected mesiotemporal substructures crucial for verbal performance resulting into both seizure freedom and good postsurgical neuropsychological outcome in some cases. Moreover, the estimation of specific hippocampal atrophy patterns might enable the clinical monitoring of verbal memory performance in patients with MCI or Alzheimer’s disease as well as the prediction of postoperative verbal memory outcome in patients with temporal lobe epilepsy.

## Data availability statement

The anonymized data supporting the conclusions of this article will be made available by the authors, upon reasonable request.

## Ethics statement

The studies involving human participants were reviewed and approved by Ethics Commission Salzburg/Ethikkommission Land Salzburg; approval number 415-E/1429. The patients/participants provided their written informed consent to participate in this study.

## Author contributions

P-ET: study concept and design, data analysis, interpretation, statistical analysis, manuscript writing, and revision. C-JM: data acquisition, interpretation, statistical analysis, and manuscript revision. MB, FZ, and KM: data analysis and manuscript revision. ET: data acquisition, analysis, interpretation, and manuscript revision. SK and AT: study concept and design, data acquisition, analysis, interpretation, and manuscript revision. All authors contributed to the article and approved the submitted version.

## Footnotes

^1^ https://surfer.nmr.mgh.harvard.edu/

^2^ https://surfer.nmr.mgh.harvard.edu/fswiki/recon-all
